# Effects of the Combination of Music Therapy and Physiotherapy in the Improvement of Motor Function in Cerebral Palsy: A Challenge for Research

**DOI:** 10.3390/children8100868

**Published:** 2021-09-29

**Authors:** Maria Jesus Vinolo-Gil, Esteban Casado-Fernández, Veronica Perez-Cabezas, Gloria Gonzalez-Medina, Francisco Javier Martín-Vega, Rocío Martín-Valero

**Affiliations:** 1 Department of Nursing and Physiotherapy, Faculty of Nursing and Physiotherapy, University of Cadiz, 11009 Cádiz, Spain; mariajesus.vinolo@uca.es (M.J.V.-G.); esteban_cf94@hotmail.es (E.C.-F.); veronica.perezcabezas@uca.es (V.P.-C.); gloriagonzalez.medina@uca.es (G.G.-M.); javier.martin@uca.es (F.J.M.-V.); 2 Department of Physiotherapy, Faculty of Health Sciences, University of Málaga, 29071 Málaga, Spain

**Keywords:** cerebral palsy, music-therapy, physiotherapy, motor function, review

## Abstract

Background: There are different therapeutic strategies such as physiotherapy and music therapy for the treatment of cerebral palsy. Intervention protocols using both therapies to unify the measurement of motor function have not been investigated. Aims and scope: To summarize the effects of the treatment of cerebral palsy through the use of both for the improvement of motor function, analyse the challenges encountered, and submit proposals for improving them. Methods: The systematic review was conducted following PRISMA guidelines and registered in the PROSPERO database (CRD42020162493). Clinical trials that described the results obtained in terms of motor function through physiotherapy and music therapy were included. Results: Eight clinical trials with 234 participants were considered with a significant improvement in motor function. Results of meta-analysis suggested improvements in gait velocity in favour of the control group for cerebral palsy (mean differences = 0.03; 95% confidence interval = 0.01, 0.04, *p* = 0.001; *I*^2^ = 97%). However, high heterogeneity was identified in the meta-analysis due to the small number of studies included. Conclusions: The combination can be effective in subjects with cerebral palsy to improve motor function, although due to the diversity of studies analysed, it is complex to extrapolate results.

## 1. Introduction

Cerebral palsy (CP) is defined as a group of permanent disorders in the development of movements and postures, provoking limitations on activity, attributed to non-progressive disturbances that occurred in the development of the foetal or infant brain. Motor disorders of CP are often accompanied by alterations to sensation, perception, cognition, communication, and behaviour due to epilepsy and secondary musculoskeletal problems [1].

CP affects approximately 1 out of 500 new-borns, with an estimated prevalence of 17 million people worldwide [2], which establishes it as the most common motor disability during childhood [3].

Physical therapy (PT) plays a key role in its treatment through several therapeutic interventions that achieve improved physiological and functional outcomes [4]. Based on recent evidence, current goal-oriented functional approaches are considered effective, although more research is needed to determine the best ways to achieve even more improved functional outcomes in children with CP [5]. Music therapy (MT) might be a new avenue that, in combination with PT, would help to improve motor function in patients with CP [6,7].

In the words of Jackson and his collaborators, "Music therapy is a paramedical specialization based scientifically on clinical–therapeutic methods and establishes a working methodology and a series of techniques with the objective of promoting positive cognitive, physical, mental, and social changes in individuals with health or behaviour disorders" [8]. Through the use of rhythmic, harmonic, and melodic sounds by means of improvisations, musical compositions, reproduction of sounds, and other techniques, an improvement in communication, expression, organization, learning, and mobilization is achieved by also obtaining a rehabilitative effect [9,10].

Several studies have demonstrated the positive effect of MT on numerous conditions, such as cancer [11], burns [12], and cognitive deterioration [13], on diverse factors, such as breathing, blood flow, heart rate, blood pressure, acceleration of the metabolism, and oxygenation and on different emotional states, such as anguish, anxiety, tension, stress, and fear [14]. MT has also been used in people with CP in order to determine its influence on muscle tone [15,16,17].

Bean and Oldfield (2001) performed a series of exercises for the development of specific movements in children with CP, creating musical activities for the development of functional skills [18]. This active work was also used by Lasse Hjelm, the author of the functionally oriented music therapy (FMT) method, who commented: "music therapy focused on human body functions is often indicated in motor impairments and is highly applicable in people with CP” [19]. Another example of the application of active musical activities for the development of motor skills in people with CP was carried out by Hatampour and his collaborators [20].

In terms of functional capacity, certain authors have advanced the traditional concepts of music and education, such as Richardson, who adjusted Zoltán Kodály’s methodology for children with disabilities, using more concrete techniques that helped the development of motor skills, such as manual signal tests [21], an adaptation of Orff Schulwerk’s methodology, or the Suzuki method [22]. Significant results [23] were also found through the use of music to motivate patients during physiotherapy sessions.

At the beginning of the 1990s, the first clinical collaborations between music therapists and physiotherapists began, leading to the beginnings of neurological music therapy (NMT). NMT is an evidence-based system of clinical interventions or techniques for retraining the sensory–motor area, speech and language, and cognitive functions that may be impaired following an injury of neurological origin [24]. Additionally, it can promote neuroplasticity by motor control learning through therapeutic instrumental music performance (TIMP) [25].

Other music therapy techniques have also been used, such as patterned sensory enhancement (PSE), which uses musical elements to translate the components of a movement into sound patterns in order to give temporal, spatial, and force cues about the movement to improve its execution [26], or rhythmic auditory stimulation (RAS), which uses rhythmic sensory stimuli to improve motor control in rehabilitation [27].

People with CP have affected gross motor function and manual function but may be able to integrate rhythm and movement and use musical instruments in their therapy, taking advantage of all the benefits of this technique for neuroplasticity and motor control [28]. Alves-Pinto et al. (2015) showed that functional rehabilitation in people with CP is not due to chance, since MT introduces changes in the internal mechanisms underlying motor function [29].

Several studies can also be found on elderly people [30], patients with Parkinson’s [31,32], or patients with cerebrovascular accidents [33] that analyse how a combination of PT and MT achieves improvements in motor function. However, there are few trials in which they are used for the treatment of CP [28].

Based on the above, we set the aim of learning the effects of PT in combination with MT in the treatment of motor function in people with CP according to the consulted bibliography, analysing the difficulties encountered in the study of this type of multivariate interventions and submitting proposals for improving them.

## 2. Materials and Methods

### 2.1. Design

A systematic review and meta-analysis were conducted and recorded in PROSPERO (CRD42020162493) using the Preferred Reporting Items for Systematic Reviews and Meta-analysis (PRISMA) [34].

All analyses were performed using data from previously published studies, and therefore, no ethical approval or patient’s consent was required.

### 2.2. Search Strategy

A literature review was conducted to identify clinical studies treating people with CP with a combination of PT and MT. The following databases were used: Web of Science, PubMed, CINALH, Medline through EBSCOhost, PEDro, ProQuest, Ovid, Scopus, Cochrane Plus, ABI Inform/ProQuest, JSTOR, EBSCO, ScienceDirect, Emerald, PROSPERO, SSRN, and PsycInfo. In addition, grey literature (TESEO, OpenGrey, and Grey Literature Database) and music therapy conference proceedings were searched.

Two reviewers ran several searches using combinations of the following keywords: “cerebral palsy”, “physical therapy”, “music therapy”, “music”, and “function” with the Boolean operators “AND” and “OR”. The initial search was carried out in December 2019 and was completed with a new search to update the review to December 2020.

An example of one of the searches carried out in PubMed is shown in Appendix A.

### 2.3. Selection of Studies

All studies identified in the searches were assessed by two reviewers to decide whether to include them. The results were initially selected according to title and abstract. After reading the full text and considering it suitable, they were assessed for their relevance according to the inclusion and exclusion criteria. Any disagreements were resolved by discussion between both reviewers until a consensus was reached. All references resulting from the search strategy were imported into the Mendeley reference manager, and duplicates were removed.

### 2.4. Eligibility Criteria

Eligibility criteria were based on the PICOS framework [27] as follows: Participants (people with PC, without age limit, who were able to follow verbal instructions and cooperate during the study, with no hearing loss), Intervention (any treatment intervention that uses MT in combination with PT), Comparison (intervention only with physiotherapy, only with music therapy, or no treatment), and Outcome (motor function: understood as the ability to plan and carry out voluntary movements [35]).

Regarding the design of the study, clinical trials were considered.

For all eligible trials, data extraction was independently completed by two authors and was cross-checked for consistency by a third author. The primary authors of eligible trials were contacted when information was considered to be missing for either the quality assessment or the data extraction process.

We included clinical trials that described the results obtained in terms of motor function through PT and MT in the treatment of CP. No limitations were made in terms of language, date, patient’s age, or the type of musical intervention. This review required the study results to clearly measure the effects of the combination of PT with MT on motor function; therefore, we excluded research where no quantitative methods were used. There were no limitations in terms of comparing interventions, which could include, besides generic music, other types of music or alternative sounds. There was also no limitation on the physical therapy treatment used. Studies were accepted in which NMT was used even if no physiotherapists were involved.

### 2.5. Assessment of Methodological Quality

The assessment of the methodological quality of the clinical trials found in our search was performed using the PEDro scale (Physiotherapy Evidence Database) and was carried out independently by two reviewers. The PEDro scale scores 10 items: random allocation, concealed allocation, similarity at baseline, subject blinding, therapist blinding, assessor blinding, >85% follow-up for at least one key outcome, intention-to-treat analysis, between-group statistical comparison for at least one key outcome, and point and variability measures for at least one key outcome. Items are scored as either present (1) or absent (0), and a score out of 10 is obtained by summation. The scale includes an additional item (eligibility criteria) to evaluate the external validity, but the score is not counted. It is based on the list developed by Verhagen [36] and evaluates the internal validity of randomised controlled trials. A study with a PEDro score of 6 or above is considered level 1 evidence (6–8: good; 9–10: excellent), and one with a score of 5 or less is considered level 2 evidence (4–5: low; <4: poor) [37]. Disagreements between authors were initially resolved by discussion and then by consultation with a third reviewer.

The risk of bias was calculated for each study using the Cochrane Collaboration´s tool [38], referring to the following types of bias: selection bias, performance bias, detection bias, attrition bias, reporting bias, and other bias. The risk of bias and the study quality were calculated by two reviewers. In case of doubt, the final decision was determined through discussion including a third reviewer.

### 2.6. Statistical Analysis

A meta-analysis was applied to compare changes in the effect size (post-intervention and pre-intervention) between the intervention and the control group. For the meta-analysis, the mean differences or standardised mean difference (MD) was calculated along with the 95% confidence interval, with the significance level set to *p* < 0.05. Heterogeneity was determined by the chi-square test and the *I*^2^ statistic. The results of all the subgroups included in this meta-analysis were represented in forest plots. The statistical analyses and the sensitivity in the meta-analysis were carried out with the statistical software Review Manager 5.4.1 (The Cochrane Collaboration 2021).

The Begg and Egger´s tests were used to determine the presence of publication bias. Publication bias is taken as a reference when *p* < 0.1. The EPIDAT 4.2 programme was used for its calculation [39].

## 3. Results

### 3.1. Selection of Studies

The search strategy was carried out on 17 databases, from which we obtained 151 articles. Of these, 5 belonged to Web of Science, 5 to PubMed, 3 to CINALH, 1 to Medline, 6 to PEDro, 44 to ProQuest, 22 to Ovid, 6 to Scopus, 3 to Cochrane Plus, 1 to ABI Inform/ProQuest, 18 to JSTOR, 0 to EBSCO, 26 to ScienceDirect, 1 to Emerald, 0 to PROSPERO, 0 to SSRN, 8 to PsycInfo, 2 to TESEO, 0 to OpenGrey, 1 to Grey Literature Database, and 1 to Music Therapy Conference Proceedings.

**Figure 1:**
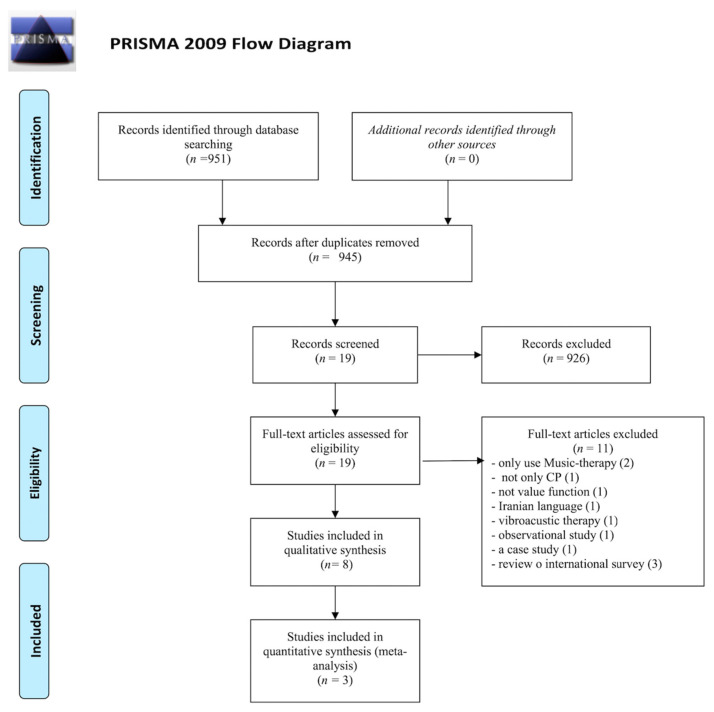
Information flowchart of the different phases of the systematic review, according to the Preferred Reporting Items for Systematic Reviews and Meta-Analyses (PRISMA) guidelines.

After filtering by eligibility criteria, focusing on the efficacy of the treatment on cerebral palsy to improve the motor function through MT and PT and eliminating duplicate articles, 30 documents were obtained. After reading them, eight articles were judged to be valid for the review. The main steps related to the bibliographic search phase are presented in Figure 1 using a flowchart.

### 3.2. Study Design and Characteristics

Eight studies with a total of 234 participants diagnosed with cerebral palsy aged between 4 [25] and 52 [40] years were included in this systematic review. The main findings of the systematic review are presented in Table 1. The type of cerebral palsy, the average age of the patients, the “motor function” outcome, as well as the most relevant results are described in Table 1. Table 2 shows intervention details.

Different types of CP were studied: spastic diplegia [26,45], spastic hemiplegia [40,44], bilateral spasticity [42,43], spastic CP [41], and severe bilateral CP [25].

With respect to the motor function outcome, the following were analysed: kinematic parameters and temporal data (such as cadence, velocity, step length, trunk flexion angles, centre of mass movement, and symmetry) [26,41,43,44], GDI step deviation index [42,43], normalized jerk index (NJI) [26], lift and walk test [40], 10 meter gait test [40], Berg balance scale [40], TUG [40], balance [40], gross motor function measure (GMFM) [45], paediatric evaluation of disability inventory (PEDI) [45], STS [45], upper limb functionality [25], Chailey’s ability levels, and Communication Function Classification System (CFCS) [25].

As for the interventions used, the studies analysed present different characteristics in terms of the performance within the treatment sessions. The differences reside in previous trainings, duration of treatment or session, number of sessions, and number of participants, all of them variables for measuring function and performance of the techniques, either by repetition or by working time.

Working sessions during treatment vary across studies, being at least one session [26,42], and at most 60 sessions [44], with studies normally lasting from 3 to 12 weeks. The duration of the sessions varied between 30 min [41,43,46] and 60 min [44], and the frequency varied between twice a week [40] and five times a week [41,44].

In terms of PT techniques, the most frequent was gait retraining [41,42,44,47], followed by neurodevelopmental therapy (NDT) [25,43,44]; two articles used resistance techniques [26,45], and one used technique for sports training with a ball and a disc [40].

With respect to MT techniques, rhythmic auditory stimulation (RAS) was used in three of the trials [40,42,43], sensory enhancement pattern (PSE) was used in another two [26,45], a pedometer with music was also used [44], and the last one was based on a programme of instrumental music performance using neurological music therapy (TIMP) [25]. Five articles used an electronic keyboard [25,26,42,43,45].

Some cooperative work has been carried out between specialized physiotherapists and music therapists to adapt the different musical pieces to the patients’ characteristics so that they could achieve the goals proposed.

As regards the statistical evaluation in the studies analysed, for kinematic parameters of the pelvis and hip joint evaluated in the sagittal plane, Kim et al. [42] found significant changes with RAS: anterior pelvic tilt at initial contact was significantly ameliorated by RAS (15.09 ± 9.78 degrees, vs. 17.27 ± 7.64 degrees without RAS; *t* = 2.874, *p* = 0.008 by paired t-test), and angles of maximal and minimal hip flexion during a gait cycle were both significantly reduced with RAS (49.20 ± 10.99 degrees to 47.82 ± 10.87 degrees in maximal flexion, *t* = 2.373, *p* = 0.025; 9.17 ± 10.99 degrees to 7.52 ± 11.88 degrees in minimal flexion, *t* = 2.468, *p* = 0.020). However, in a later study [41], RAS aggravated maximal internal rotation in the transverse plane (*p* < 0.05). With respect to the findings in the knee, Peng et al. [26] achieved a small but significant difference in peak knee extensor power per limb (*p* = 0.04), with an effect size of 0.23, and total extensor power per limb, with *p* = 0.03 and an effect size of 0.35.

About gait tests, Efraimidou et al. [40] revealed a statistically significant main effect of time or group in gait time (s) (F1,8 = 13.60, *p* = 0.006, η^2^ = 0.630) in the TUG-test and on normal gait speed (m/s) (F1,8 = 8.53, *p* = 0.019, η^2^ = 0.516), but not on fast gait speed (m/s) (F1,8 = 4.84, *p* = 0.059, η^2^ = 0.377) in 10MWT. The ANOVA repeated measurement test revealed a statistically significant main effect of time or group on Berg Balance score (F1,8 = 18.01, *p* = 0.003, η^2^ = 0.692) in BBS.

Previously, Hamed et al. [44] noted that velocity was 0.68 ± 0.09 m/s (0.26 ± 0.07 change score) for the study group and 0.42 ± 0.11 m/s (0.060 ± 0.05 change score) for the control group (*t* = 6.2) (*p* < 0.0001); stride length was 0.52 ± 0.07 metres vs. 0.34 ± 0.07 metres for control (*t* = 6.25) (*p* < 0.0010); however, cadence was much less significant at 124.3 ± 4.3 steps/min (−5.8 ± 2.1 change score) for the study group and 128.7 ± 4.1 steps/min (−0.86 ± 0.05 change score) for the control group (*t* = 2.8) (*p* < 0.008). Meanwhile, Kwak et al. [41] observed increased stride length (*t* = −3.109, *p* = 0.014) and velocity (*t* = −3.029, *p* = 0.016) in the therapist-guided training group.

On the other hand, Wang [45] showed GMFM score means at four time points and observed in both groups significant main time effects of GMFM scores in Dimensions D (F = 8.9–32.2, *p* ≤ 0.005) and E (F = 6.4–16.4, *p* ≤ 0.016) and the Goal Dimension (F = 12.7–28.3, *p* ≤ 0.001). Additionally, significant group by time interactions were found in Dimension D at T1 (after 6 weeks of training) (*p* = 0.004, mean adjusted difference = 3.6, ES 0.54) and T2 (at 6 weeks) (*p* = 0.004, mean adjusted difference = 3.8, ES 0.54), with greater improvements in the PSE group than in the no-music group. However, the music effects did not maintain at T3 (at 12 weeks following the end of the training) (*p* = 0.06, ES 0.34). For the goal score, significant interactions were found at T1 (*p* < 0.001, ES 0.7), T2 (*p* = 0.004), ES 0.54, and T3 (*p* = 0.013, ES 0.46). The Functional Skills Scale scores of the PEDI mobility domain increased in both groups from T0 (at baseline) to T1, T0 to T2, and T0 to T3, resulting in significant main effects of time (F = 9.4–12.6, 0.001 ≤ *p* ≤ 0.004) [43].

Values of STS 1RM increased in both groups, resulting in a significant main effect of time at all time points (F = 11.3–15.6, *p* ≤ 0.002), but no interaction was found (*p* = 0.06–0.15) [45].

Finally, the only study that did not assess the lower limb, Marrades et al. [25], found significant differences in the Total Chailey Test (*p* = 0.002), arm and hand position section (*p* = 0.027), activities (*p* = 0.007), and locomotor stages (*p* = 0.008).

### 3.3. Assessment of Methodological Quality

Table 3 shows the evaluation of the methodological quality according to the PEDro scale (its average is 5.5). We found four studies [42,43,44,45] with Level 1 evidence (good, 50%, 4/8); two studies [25,40] with a score of 5, which is considered Level 2 evidence (acceptable; 25%, 2/8); and two studies [26,41] that had a score of 4, which is considered Level 3 evidence (poor; 25% 2/8). Trials were of sufficient methodological quality if they had a score of at least 5 out of 10 points. This was considered because tests with a score close to 4 do not employ a triple-blind methodology (i.e., patient, assessor, and treatment). In only one study, the assessment was carried out by the same paediatric physiotherapist who was blinded to the intervention group [25].

### 3.4. Quantitative Analysis

**Figure 2:**
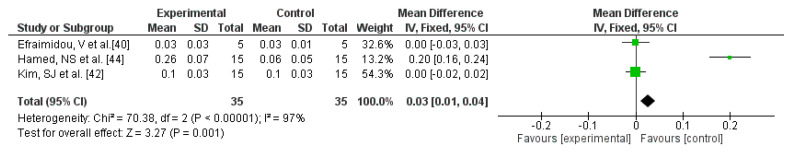
Forest plot for overall studies about gait velocity.

Gait velocity was only compared with no intervention in three studies [40,43,44]. The results of the meta-analysis suggested improvements in gait velocity in favour of the control group for cerebral palsy (MD = 0.03; 95% CI = 0.01, 0.04, *p* < 0.01; *I*^2^ = 97%). However, high heterogeneity was identified in the meta-analysis due to the small number of studies included. The corresponding forest plot is shown in Figure 2.

**Figure 3:**
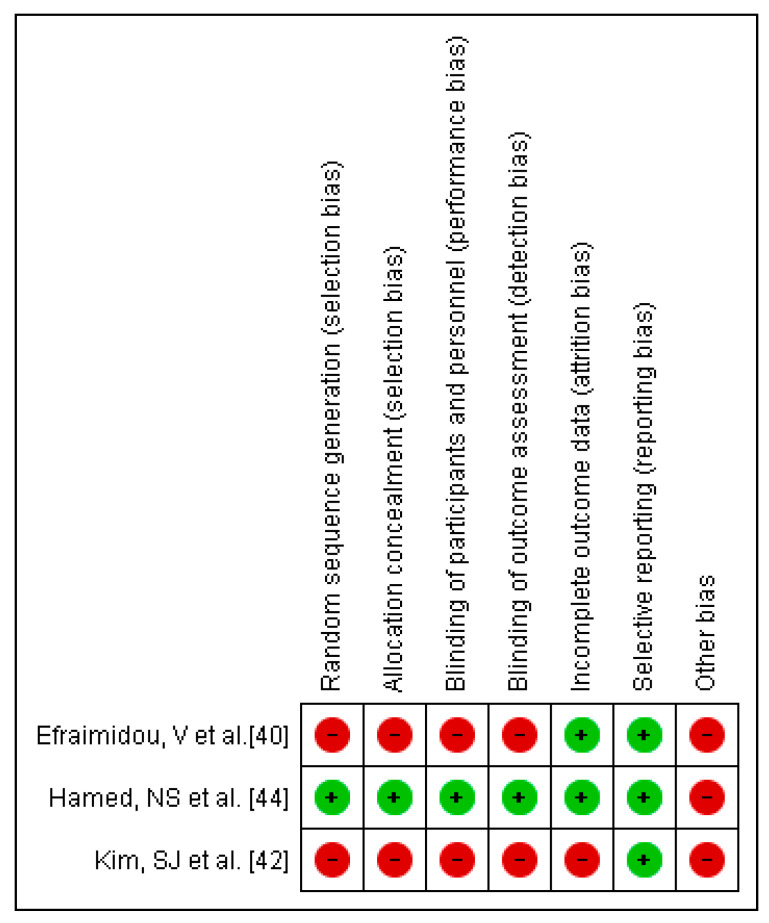
Risk of bias graph.

**Figure 4:**
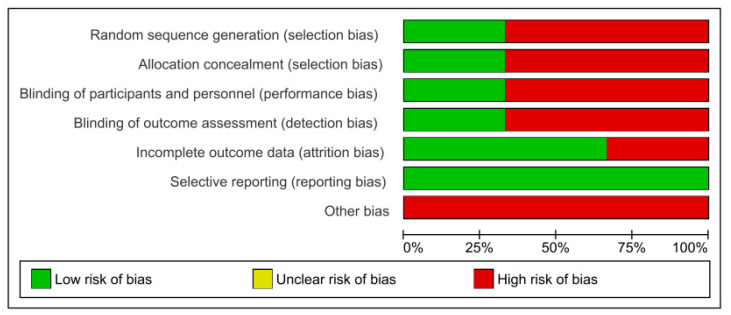
Risk of bias summary.

The results of the risk of bias can be observed in Figure 3. It should be noted that the risk of bias is high for the studies included in the meta-analysis. With respect to other bias, none were low-risk. In relation to reporting bias, all of them were high-risk (Figure 4).

The sensitivity of the meta-analysis indicates that there is no statistically significant change if we eliminate Efraimidou [40] and Kim´s studies [42]. That is, the results of the meta-analysis still support the control group. However, when the Hamed´s study [44] is removed, there are no statistically significant results for either group.

Finally, Begg’s test value (*p* = 1.00) and Egger’s test value (*p* = 0.97) indicate that there is no publication bias.

## 4. Discussion

This systematic review summarizes the findings collected in the literature regarding the effects of MT in combination with PT for the treatment of CP and the improvement of motor function. The efficacy of the combined use of MT and PT to improve motor function in the treatment of CP has been viewed positively, as a significant improvement in motor function has been obtained in all the studies. The combination improved stride length, velocity, symmetry, cadence, step length, knee extension power, balance, upper limb position, and locomotor stages. According to the results of the meta-analysis performed, it did not improve walking speed.

However, we have found some challenges associated with assessing multivariable interventions like PT and MT, making it impossible to obtain conclusive statistical results.

### 4.1. Selection of Studies

Great difficulties were encountered in the bibliographic search because few studies have analysed the interaction of both treatment modalities. We had to extend our search to more databases, grey literature, and conference proceedings, without setting limits on dates or language. Additionally, the search limits, such as the age of the participants or the type of intervention, had to be extended. Despite this, only eight articles could be selected. However, these eight selected articles present great variability. This represents a new challenge in physiotherapy: the evaluation of multivariate interventions of physical and music therapies due to the great heterogeneity of the interventions in the studies found.

### 4.2. Characteristics of Studies Included

Firstly, the samples were small. This is the main problem in this type of research, sometimes due to the age of the participants or the long duration to achieve effective results [26]. The difficulty of accessing a sufficient sample means that effective statistical analysis cannot be carried out.

Secondly, the samples were also very heterogeneous in terms of age and level of severity. In terms of age, in some investigations the samples were composed of children [25,26,44,45], others were composed of adults [40,42,43], and in one study, there were both children and adults [41]. Bearing in mind that age can be a determining factor in possible recovery, it should also be taken into account that plasticity through specific motor tasks is seen more clearly during childhood; however, it can be higher when practice is continued throughout life [48]. It should be noted that surgery was considered by four of the eight studies reviewed as an exclusion criteria. Two of the trials took into account that neurological or orthopaedic surgery had not been performed within the last 12 months [41,44], and the other two documents had as exclusion criteria that it had not been performed in the last 6 months [45], with one of them considering lumbar spine surgery [26]. It is also worth noting that four of the studies mentioned that training can be extended to those dependent on assistive devices [26,43,44,45].

With regard to the level of severity, some studies were on children who could stand and even walk [40,41,42,43,44] or sit and stand [45], while others’ participants had more severe pathology [25]. In one of the studies, the control group was even made up of healthy people [42]. It should be mentioned that the studies using the STS test [26,44] highlight the need to use assistive devices to stand up [26] and to detect horizontal movements [44]. Nevertheless, Wang’s study points out the importance of adapting them to strength and resistance guidelines [45].

There was also great variability in terms of their classification. One of the difficulties of the studies carried out on CP lies in the heterogeneity of this clinical group, as the patients differ greatly in the symptoms they present [49]. In our review, most studies dealt with spastic CP, although most studies provided very general data and one did not even specify the type [25]. There should be a more uniform criterion for their classification. The most accepted classification is that of clinical manifestations, in relation to the number of affected limbs, muscle tone, and altered mobility. Among these, we have spastic hemiparesis and spastic quadriparesis. In addition, varieties of quadriparesis such as hypotonic and choreoathetotic varieties can occur. There are also spastic diparesis and extrapyramidal, atonic, and ataxic cerebral palsy [50].

Apart from specifying the symptomatology and its corporal affectation, it would be advisable to complete the description of the patients using scales to evaluate their motor functions, manual function, and communication [51]. As noted in this review, motor function has been measured in different ways, with a lack of homogeneity and no established criteria on how to measure it. Even so, two main groups of study can be distinguished. One group analyses the motor function in the upper limb [25], and the other analyses the motor function in the lower limb [26,40,41,42,44,45].

It may be appropriate to use different tools that would help to unify the criteria. Motor function could be classified according to the functionality criteria of the Gross Motor Function Measure (GMFM-88), which is a measurement scale designed to see the changes in gross motor skills of children with cerebral palsy. There are two versions composed of 66 and 88 elements subdivided into five dimensions: (A) decubitus and rolling, (B) sitting, (C) crawling and kneeling, (D) standing, and (E) running and jumping. It is graded on a scale from 0 to 3 points, where 0 means the participant cannot start the task, and 3 means they are able to perform the task completely, without judging the quality of the patterns. The final result is given in percentages, which allows the overall state of gross motor skills to be quantified more precisely [52].

To assess manipulative ability in children with CP aged 4–18 years, the Manual Ability Classification System (MACS) scale can be used [53]. It also has five levels, level I being the most functional and V the most severe. It could be completed with the Communication Function Classification System (CFCS) [54]. This classification is for the level of communication, with level I indicating effective communication and level V meaning inconsistent communication.

There is also no homogeneity in terms of frequency, duration of treatment, or number of sessions. Our results vary greatly. Kwak proposed a duration of 20 min so as not to tire the patient, twice a week, and as for the total duration of the treatment, it would depend on the objective [41]. In one of the articles, the treatment was performed twice a week, [40] in another five times a week [41,44], and in another one it was not even specified [25]. The duration of the treatment ranged from 1 day [26] to 3 months [44], and the sessions lasted between half an hour [41,43] and 1 hour [44].

It has also been a challenge to find documents that establish clear limits to the music therapist’s or physiotherapist’s input on motor function. In almost all the studies, it was not clear whether physiotherapists were participating in the trial, although interventions were being made within their remit. Only in three studies [25,43,44] was the physiotherapist mentioned, although the techniques used were not exactly specified. Two of them used neurodevelopmental therapy/Bobath [43,44], and the other did not even specify the type of physiotherapy used [25]. In reference to the TM techniques, they were better detailed. However, according to Tiburcio, it should be specified whether the strategy to be used is receptive TM or active TM [17].

NMT was used in all the selected articles except the one by Hamed et al., where a pedometer was used [44]. Within the NMT, techniques such as RAS [40,42,43], PSE [26,45], and TIMP [25] were used. Other authors have concurred on the use of the latter technique for the treatment of the upper limb [28,46]. There is evidence that the rhythm used in these techniques facilitates motor activities, improving gait and modulating muscle tone in patients with neurological alterations [55].

The current scientific literature is looking for new options for the treatment of CP within PT studies, although not in combination with MT. In the case of MT, the use of apps [56], auditory feedback devices [57] and finger exercises with a keyboard [58] or a piano jacket [59] have been proposed, while in the area of PT, robot-assisted rolling therapy [60,61], the use of robotic exoskeletons [62], non-invasive electrical stimulation [63], robots [64] or simulators in hippotherapy [65], and walking assistance robots [66,67] have been proposed.

In the case of walking and upper limb function, the scientific evidence points to RAS [68], TIMP to simulate non-musical movements of daily life, and PSE to perform movements typical of daily life activities that after repetition are learnt, adopted, and integrated [48]. On the other hand, PT is an essential component in the treatment of patients with CP [69], and the music therapist can offer a structured and systematized way to perform PT exercises respecting speed and duration, facilitating the work of the physiotherapist and the patient [70]. We believe that more research should be done on these two disciplines used together to increase the potential of each one separately. There should be close collaboration between the two interventions but limiting their performance within the study, and joint intervention protocols could be drawn up for interdisciplinary teams where music therapists and physiotherapists work together, where each side could support the other, with the common objective of improving the patient’s motor function [41].

It is important to consider the type of CP in the choice of therapy. People with cerebral palsy do not respond equally to music, and individual responses to musical stimulation should be observed and evaluated, as they respond specifically to different types of musical stimuli [71]. In the literature consulted, only studies on spastic CP have been performed, but we believe it is interesting that studies are being carried out on other types of CP. It has been observed that in people with athetoid CP, music that generates variations in mood can cause fluctuations in tone between hypertonia and hypotonia [28].

Finally, although no conclusive results can be extrapolated from the above, the effect of the combined use of MT and PT to improve motor function on the treatment of CP has been seen as positive in the literature considered, as a significant improvement in motor function was obtained by all of the studies. There were improvements in kinematic and temporal parameters of gait [26,40,41,42,43,44], balance [40], functionality scales such as GMFM [45], and Chailey’s ability levels [25].

Therefore, it would be relevant to combine both therapies in the treatment of CP. However, we need more standard interpretation models to measure functionality and additional standardized data to validate the existing literature and thus be capable of achieving a treatment protocol. This will allow us to achieve an optimal therapeutic treatment [26].

### 4.3. Assessment of Methodological Quality

In most of the articles, the subjects were not randomly assigned. Only the studies by Hamed et al. [44] and Wang et al. [45] fulfilled this condition. Only 12% of the articles had the evaluators blinded [45]. Randomisation and blinding of assessors should also be ensured across studies. This also highlights the need for the authors to use the same measurement instruments, because it was not possible to compare studies statistically because different measurement methods were used. It could be recommended for clinical trials to adopt the CONSORT rules [72].

As for the methodological quality of the studies, only 40% were of acceptable quality according to the PEDro scale. The criteria that were least met were Items 5 and 6. This is because these types of interventions are very difficult to blind, and there is an impossibility of triple-blinding. However, we believe that the rest of the criteria can be improved in further research.

### 4.4. Limitations

As for limitations, it was difficult to find literature on the subject, and what little was found was very heterogeneous and of poor methodological quality. With such small samples and the heterogeneity found in the results, it was difficult to perform a meta-analysis with extrapolable results; therefore, it would be appropriate to analyse which aspects would need to be improved or considered in future research.

### 4.5. Implications in the Field of Physiotherapy and Research

It should be mentioned that in this study, an exhaustive search of all the literature was carried out, including the grey literature, so it could serve as a starting point for the unification of certain criteria applied in physiotherapy and music therapy research.

It will be necessary to conduct clinical trials to identify which specific factors of therapy have a greater weight in achieving a positive outcome. A common interdisciplinary work between music therapists and physiotherapists would be appropriate. It would be necessary to see the cerebral palsy patient as a whole and not to be selective in their treatment, analysing only the upper or lower limb.

We encourage researchers to perform high-quality clinical trials using larger sample sizes and greater homogeneity in terms of the types of CP, devices used, and intervention protocols of both MT and PT, considering the frequency and intensity, as well as to unify criteria in terms of how to measure motor function.

In addition, we emphasize the need for clinical trials that prove the efficacy of this combination in order to provide greater scientific support in the treatment of patients with CP.

## 5. Conclusions

In conclusion, based on the results obtained in the present review and meta-analysis, we can say that physiotherapy in combination with music therapy can be effective in subjects with cerebral palsy to improve motor function in a general way, allowing them to perform voluntary movements more easily. This multivariate intervention improved stride length, velocity, symmetry, cadence, step length, knee extension power, balance, upper limb position, and locomotor stages. However, it was not effective on walking velocity. Although, due to the diversity of studies analysed and the number of articles included in this study, no firm conclusions can be drawn.

In general, a positive impact of physiotherapy and music therapy on cerebral palsy is expected, but future clinical trials are needed that use larger sample sizes and present greater homogeneity in terms of the types of CP, devices used, and intervention protocols of MT and PT, as well as unify criteria in terms of how to measure motor function.

## Figures and Tables

**Table 1:** Results of studies included within the systematic review.

Study	N Age/Mean (SD) Age Range (Years)	Type of PCI	“Motor Function” Outcome	t/w min Weeks	Results
Kwak [41] (NRCT)	*n* = 25 Con: 9 Exp1: 9 Exp2: 7 6–20	Spastic cerebral palsy	14-meter walkway Gait parameter (cadence, stride length, velocity, symmetry)	5 t/w 30 min 3 weeks	Exp1: improved stride length (*t* = −3109, *p* = 0.014), velocity (*t* = −3029, *p* = 0.016) and symmetry (*t* = −3029, *p* = 0.016)
Kim et al. [42] (NRCT)	*n* = 44 Con: 30 healthy people Exp: 14 with CP 25.64 ± 7.31	Bilateral spasticity	Temporal and kinematic gait parameters GDI Asymmetry	1 session	Kinematic changes of pelvic and hip movement, and GDI was significantly changed with RAS (*p* < 0.05) Improved temporospatial asymmetry in household ambulators
Kim et al. [43] (RCT)	*n* = 39 Con: 19 27.3 (2.5) Exp: 20 27.3 (2.4)	Bilateral spasticity	Temporal and kinematic parameters of the gait GDI	3 t/w 30 min 3 weeks	Exp: improved cadence, velocity, step length, stride length (*p* < 0.05), previous pelvic tilt, hip flexion (*p* < 0.05), GDI (*p <* 0.05) Con: improved Ri and Re (*p* < 0.05).
Peng et al. [26] (RCT)	*n* = 23 8.7 (2) Con: NA Exp: NA	Spastic diplegia	Kinematic parameters Movement time NJI Flex trunk angles COM address	1 session	Maximum knee extension power (*p* = 0.009), >total extension power (*p* = 0.015), >COM fluidity (*p* = 0.01), <movement time (*p* = 0.003), and remained without music
Hamed et al. [44] (RCT)	*n* = 30 Con: 15 7.07 (0.82) Exp: 15 Exp: 7.03 (0.76)	Spastic hemiparesis	Running speed Stride length Cadence Cycle time	5 t/w 60min 12 weeks	Exp: improved velocity (*p* < 0.0001) and cadence (*p* < 0.008) Exp: velocity 0.68 ± 0.09 m/s Con: 0.420 ± 11 m/s for control group (*t* = 6.2) (*p* < 0.0001) Exp: cadence 124.3 ± 4.3 steps/min Con: 128.7 ± 4.1 steps/min (*t* = 2.8) (*p* < 0.008)
Efraimidou et al. [40] (RCT)	*n* = 10 Con: 5 38.80 (12.28) Exp: 5 35.20 (13.01)	Spastic hemiparesis	TUG Lift and walk test MWT Berg’s Scale Static and dynamic balance with EPS platform	2 t/w 50 min 8 weeks	Exp: improvement in gait and balance (*p* ≤ 0.05) Improvement in gait time (s) (F1,8 = 13.60, *p* = 0.006, η^2^ = 0.630), in normal gait speed (m/s) (F1,8 = 8.53, *p* = 0.019, η^2^ = 0.516), but not in fast gait speed (m/s) (F1,8 = 4.84, *p* = 0.059, η^2^ = 0.377) Statistically significant differences in intervention group between the two measurements regarding static and dynamic balance score (*t* = −8.63, df = 4, *p* = 0.001)
Wang et al. [45] (RCT)	*n* = 45 Con: 24 9 (1.99) Exp: 21 8.98 (2.61)	Spastic diplegia	GMFM PEDI STS	3t/w 6 weeks	Exp: improvements in gross motor function capacity (*p* < 0.05) maintained 6–12 weeks (*p* < 0.13). No improvements in daily functioning, strength, and walking speed.
Marrades-Caballero et al. [25] (crossover)	*n* = 18 10 (6)	Severe bilateral	UL functionality Chailey skill levels	16 weeks	Improved Chailey Levels of Ability (*p* = 0.002): “activities” section (*p* = 0.007), “arm and hand position” section (*p* = 0.027) and locomotor stages (*p* = 0.008). Persisted for 4 months.

SD: standard deviation; Con: control group; Exp: experimental group; NRCT: nor randomized controlled trial; RCT: randomized controlled trial; GDI: step deviation index; NA: not available; NDT: neurodevelopmental therapy/Bobath; t/w: times per week; MRI: maximum repetition; Ri: internal rotation; Re: external rotation; STS: sit to stand test; COM: centre of mass; NJI: normalized jerk index; TUG: timed up and go; MWT: 10-meter walk test; BBS: Berg’s balance scale; GMFM: gross motor function measure; PEDI: paediatric evaluation of disability inventory; UL: upper limb.

**Table 2:** Type of intervention.

Study	Intervention
Kwak et al. [41]	Con: conventional gait training with a physical therapist Exp1: gait training + RAS A music therapist provided verbal instructions. A drum was used to emphasize the beat. A computer speaker system played the prescribed music (4/4 m, 105–120 beats per minute). The tempo of the music was increases by 10% (2nd week) and 15% (3rd week). Depending on individual needs, the physical therapist and music therapist developed muscle strengthening exercises using PSE and TIMP. Exp2: gait training + self-guided RAS A tape and instructions were given. The researcher demonstrated how they could feel the beat and how they could walk with the prescribed music for 30 min of daily self-training.
Kim et al. [42]	-Gait training without RAS -Gait training with RAS 1. A subject walked barefoot along a 10 m walkway three times at the individual’s preferred walking speed without RAS. 2. Walking cadence (steps/min) was calculated based on the gait parameters in Step 1. 3. The tempo of metronome beats (bpm) was set to participant’s cadence obtained in Step 2. 4. RAS was provided by the music therapists, playing a simple rhythm pattern using chord progression on a keyboard with metronome beats. 5. The same chord pattern was repeated providing a continuous timing cue and period sequence for 1–2 min to help a subject adapt to RAS immediately. 6. A subject walked 10 m three times again with RAS.
Kim et al. [43]	Con: NDT. Gait training Exp: Gait training with RAS 1. A participant walked barefoot along the walkway (10 m) three times, at the individual’s preferred walking speed, before rhythmic auditory stimulation application. 2. The individual’s cadence (steps/min) was calculated based on the gait parameters in Step 1. 3. The tempo of metronome beats (bpm) was set to the participant’s cadence obtained in Step 2. 4. RAS was provided by music therapists, who played a simple rhythm pattern synchronized with the beats of a metronome, using chord progressions on a keyboard.
Peng et al. [26]	Con: STS with 50% of 1RM without PSE, 8 reps Exp: STS with 50% of 1RM, 5 reps with PSE and 3 reps without PSE The individualized PSE music was composed by a music therapist with an electronic keyboard using GarageBand software on a Mac Mini at an intensity of 75 dBA, varying the tempo, harmonies, metre.
Hamed et al. [44]	Con: NDT + usual gait training -NDT: approximation of the upper and lower limbs in a regular and rhythmic manner, facilitation of righting, equilibrium, and protective reactions, training of postural stability and equal weight shift, stretching and strengthening exercises of the upper and lower limbs and back muscles. -Gait training program without pedometer Exp: same + pedometer-based gait training programme. A talking pedometer was fastened to a belt or waistband. It played seven melodies while walking or jogging, and the tempo synchronized with walking speed. The activities included walking forward, backward, and sideways between parallel bars, on a walking line, and on a balance beam; stepping forward on a stepper (stairs); and training of walking with different obstacles and on different floor surfaces.
Efraimidou et al. [40]	Con: ball and puck training program Exp: The program included gait and balance with music exercises according to RAS. -Warm-up period: stretching exercises accompanied with music tracks of 4/4 m and a tempo of 70 beats per minute -Main part: Participants walked to the rhythm veer (music tracks of 4/4, 90 beats/min). Then they continued to move with pace in a straight line for a distance of 10 m with forward, backward, right, and left steps, as well as standing on one leg with change for some seconds. -Cool-down: relaxation exercises, breathing and music (4/4, 70 beats/min)
Wang et al. [45]	Con: Loaded STS exercise with weighted body vest. 3 sets: -1st and 3rd: load at 20% of 1RM, 10 reps -2nd: 50% of 1RM until fatigue Exp: To prescribe the PSE music, loaded STS 50% of 1RM, 6 reps. The fastest three STS movements were selected as references to prescribe PSE music using an electronic keyboard and GarageBand software on a Mac (Apple Inc., Cupertino, CA, USA). Music provided cueing of the movement period. Caregivers supervised. Every 2 weeks, the music therapist adjusted musical elements according to the individual’s needs.
Marrades-Caballero et al. [25]	Con: physiotherapy (no technique specified), 1 t/w Exp: NMT sessions by two music therapists. The music was live and customized according to each patient. Small percussion instruments, Spanish guitar, keyboard, and drums were played. Two types of activities: -At the beginning, patients chose and played the musical instruments using their own movement strategies (hitting, rubbing, crashing) to generate rhythmic music patterns. Music therapists played along. -Task-specific training with varied and incremental levels of difficulty. Music therapists composed small pieces of music testing different music parameters trying to trigger forearm pronation and supination, elbow flexion and extension, and shoulder flexion. The activities were performed in a prone position or sitting in their wheelchairs and were aimed at challenging upper-limb movements and head and trunk control.

Con: control group; Exp: experimental group; NDT: neurodevelopmental therapy/Bobath; RAS: rhythmic auditory stimulation; t/w: times per week; MRI: maximum repetition; STS: sit to stand test; Rep: repetitions; PSE: patterned sensory enhancement; TIMP: therapeutic instrumental music performance; NMT: neurologic music therapy.

**Table 3:** Methodological quality according to the PEDro scale.

Author (year)	1	2	3	4	5	6	7	8	9	10	11	Total
Kwak (2007) [41]	1	0	0	0	0	0	0	1	1	1	0	4/10
Kim (2012) [43]	1	0	0	1	0	0	0	1	1	1	1	6/10
Peng (2011) [26]	1	0	0	1	0	0	0	0	1	1	1	4/10
Hamed (2011) [44]	1	1	1	0	0	0	1	0	1	1	1	6/10
Kim (2011) [42]	1	0	0	1	0	0	1	0	1	1	1	6/10
Efraimidou (2016) [40]	1	1	0	1	0	0	0	0	1	1	1	5/10
Wang (2013) [45]	1	1	1	1	0	0	1	1	1	1	1	8/10
Marrades-Caballero (2018) [25]	1	1	0	1	0	0	1	0	0	1	1	5/10

## Data Availability

Not applicable.

## Appendix A

An example of one of the searches carried out in Pubmed.

Example of a search strategy

“Physical therapy”[All Fields] AND “Music therapy”[All Fields] AND “Cerebral Palsy”[All Fields] AND ((Review[ptyp] OR Clinical Trial[ptyp]) AND (“2009/01/01”[PDAT]: “2019/12/13”[PDAT]))
